# Characteristics of Pain in Patients Diagnosed With Ehlers–Danlos Syndrome and Hypermobility Spectrum Disorders. An Observational Study

**DOI:** 10.1155/prm/6664142

**Published:** 2026-06-27

**Authors:** Mattia Morri, Morena Tremosini, Cristiana Forni, Alice Moroni, Elena Pedrini, Alessia Di Cecco, Maria Gnoli, Luca Sangiorgi

**Affiliations:** ^1^ Servizio di Assistenza Infermieristica Tecnica e Riabilitativa, IRCCS Istituto Ortopedico Rizzoli, Bologna, 40136, Italy, ior.it; ^2^ Struttura Complessa Malattie Rare Scheletriche, IRCCS Istituto Ortopedico Rizzoli, Bologna, 40136, Italy, ior.it

**Keywords:** Ehlers–Danlos syndrome, hypermobility spectrum disorders, pain, risk factors

## Abstract

**Background:**

This study aimed to describe pain characteristics in patients diagnosed with Ehlers–Danlos syndrome (EDS) or hypermobility spectrum disorders (HSD) and to identify factors associated with moderate to severe pain at diagnosis.

**Methods:**

Ninety‐six patients were retrospectively identified through the Ce.Ma.R.S. registry (2017–2022). Pain intensity was assessed at first evaluation using a Numeric Rating Scale (0–10). Patients were grouped into no/mild pain and moderate/severe pain. Clinical and demographic data were collected, and variables significant in univariate analysis were included in multivariate logistic regression.

**Results:**

At diagnosis, 49% of patients reported moderate to severe pain. Independent predictors were older age (OR = 1.06; 95% CI: 1.03–1.10), diagnosis of hypermobile EDS (OR = 6.25; 95% CI: 1.31–29.74), and headache (OR = 3.97; 95% CI: 1.14–13.82). Use of pain medications, particularly paracetamol and NSAIDs, was strongly associated with higher pain severity (*p* < 0.001).

**Conclusion:**

Pain is a common and early symptom in EDS and HSD. Older patients, those with hypermobile EDS, and those with headache are at greater risk of severe pain. Early, multidisciplinary pain management, including pharmacological treatment, physiotherapy, and headache‐specific strategies, should be prioritized to improve outcomes and reduce long‐term disability.

**Trial Registration:** ClinicalTrials.gov identifier: NCT04133272

## 1. Introduction

Ehlers–Danlos syndrome (EDS) is a heterogeneous group of disorders characterized by an alteration in the structure and function of connective tissue, leading to widespread symptoms affecting the skin, ligaments, joints, blood vessels, and/or internal organs [1, 2]. Clinical presentations may differ in severity, ranging from mild skin and joint hyperlaxity to severe physical disability and reduced quality of life, and may even lead to life‐threatening complications [2–5]. This group of connective tissue disorders includes 13 subtypes according to the latest classification [6]. The most common form of EDS is the hypermobility (hEDS) type (1 case every 5/10,000). Its specific genetic alteration etiology is unknown, but differential diagnosis is mainly based on the patient’s evaluation and clinical history according to specific diagnostic criteria [6]. Other EDS subtypes include the classical form (cEDS; ∼1:20,000–40,000), characterized by joint hypermobility and skin involvement, and the rarer vascular form (vEDS; ∼1:50,000–200,000), marked by vascular and organ fragility.

Alongside this diagnosis, the latest classification has introduced hypermobility spectrum disorder that represents a continuum of phenotypes that have similar characteristics to hEDS but do not meet all the diagnostic criteria and for this reason are classified as a spectrum of hypermobility disorders (HSD) [7–11]. Pain in these patients is a particularly relevant and common clinical manifestation both in terms of diagnosis and impact on quality of life [5, 6, 12]. With regard to hEDS, Benistan et al. [13] reported pain as being moderate/severe in 67% of patients, and all 34 enrolled patients had chronic pain. Pain began at the age of 10 and increased over time, becoming chronic around the age of 20. Several authors have compared pain in the different types of EDS. The results are discordant. Aubry‐Rozier et al. [14] showed that in the two disease groups, hEDS and HSD, there were significant differences in terms of pain, motor disorders, and bleeding. Conversely, Copetti et al. [15] highlighted that the more complex forms of the disease, irrespective of classification, hEDS or HSD, presented higher levels of pain. According to De Koning et al., in children with genetic connective tissue disorders, pain was reported in 87% of those with hEDS and in 72% of those with other EDS subtypes, including classical, vascular, and less common forms, with greater intensity observed in the hEDS group [16]. Personal characteristics, such as age, gender, and BMI, and disease presentation, Beighton score, episodes of dislocation, and affected joints are factors that have been scarcely investigated with regard to pain. Therefore, it is also necessary to evaluate the role of concomitant somatic symptoms such as headaches and depression that these patients often report, which can have a significant impact on pain and disability. Thus, the aim of this study was to describe pain in a monocentric cohort of patients affected by EDS (any subtype) and HSD at the time of diagnosis and identify personal characteristics, disease‐specific presentation, and nonspecific concomitant conditions associated with moderate/severe pain.

## 2. Method

This retrospective cross‐sectional study was conducted at a tertiary referral center for rare skeletal diseases. The study protocol was approved by the competent Ethics Committee Area Vasta Emilia Centro (Protocol No. 800/2022/Oss/IOR).

Patients evaluated at the Rare Skeletal Diseases Center (Ce.Ma.R.S.) outpatient clinic between 2017 and 2022 were retrospectively identified using the institutional disease registry. These patients underwent an initial evaluation, during which a diagnosis of EDS or HSD was confirmed according to the criteria proposed by Malfait et al. [6] and all subtypes of EDS were taken into consideration. All participants underwent a comprehensive clinical assessment carried out by a multidisciplinary team. Diagnosis was established according to clinical criteria, confirmed by genetic testing when available. All consecutively assessed patients meeting diagnostic criteria were included in the analysis.

### 2.1. Primary Outcome

Pain intensity, defined as that measured at the time of the clinical visit, was selected as the primary outcome and assessed using age‐appropriate validated instruments. Depending on age, pain was assessed using the Face, Legs, Activity, Cry, and Consolability Scale (FLACC), the Verbal Rating Scale (VRS), or the Numeric Rating Scale (NRS). Pain scores ranged from 0 (*no pain*) to 10 (*maximum pain*). For analytical purposes, pain was dichotomized into no/mild and moderate/severe using a cutoff value of 4 [17–19], corresponding to the established threshold between mild pain (0–3) and at least moderate pain (≥ 4), commonly used in clinical research to identify clinically relevant pain [17–19]. Pain was also described in relation to its location (spine, upper limbs, or lower limbs) and whether it was located in a joint following dislocation.

### 2.2. Variables

A predefined set of demographic and clinical variables was collected at baseline. The patient’s personal data included age, sex, height, weight, and body mass index (BMI). Genetic connective tissue disorders were classified as HSD or EDS, with the latter further differentiated into hEDS and other subtypes, including classical, vascular, and other less common forms. Clinical data included the Beighton score [20, 21], the presence of prolapses and/or hernias, and previous surgery. The site of joint dislocation was recorded, and if so, whether a single joint or several joints were involved and which specific joints (shoulders, jaw, hip, knee/patella, hands/fingers/wrist, and ankle).

The patients’ complete medical history was systematically reviewed from clinical records. Among all reported coexisting conditions, those considered most relevant in relation to chronic pain were extracted and classified as follows: nonspecific coexisting diseases included depression and anxiety disorders confirmed by medical diagnosis, previous diagnosis of fibromyalgia with conditions of widespread pain and fatigue, neuropathic disorders, headaches, and cardiovascular diseases (hypertension and stroke).

Finally, pain management strategies were recorded, including ongoing pharmacological therapy and conservative treatments such as physiotherapy aimed at muscle strengthening and general physical activity.

### 2.3. Data Collection

Clinical data were extracted from medical records and the disease registry and compiled for analysis. Data were entered into a single pseudoanonymized electronic database for statistical analysis. The research nurse from the Rare Disease Genetics clinic was responsible for data collection and management.

### 2.4. Sample Size

Given the low prevalence of these conditions, all eligible patients evaluated during the study period were included. Assuming a convenience sample of 100 subjects and considering a proportion of patients with moderate/severe pain of 0.67, it is possible to calculate the precision of the estimate with a 95% confidence interval ranging from 0.58 to 0.76.

### 2.5. Statistical Analysis

Descriptive statistics were used to summarize pain outcomes and clinical variables, applying appropriate measures of central tendency and dispersion for continuous variables. Absolute frequencies and percentages were used for categorical variables. The values of interest were reported in relation to the overall enrolled population, the group with no/mild pain, and the group with moderate/severe pain. To investigate the association between pain symptoms (absent/mild vs moderate/severe) and the collected variables related to personal characteristics and specific and nonspecific clinical characteristics of the disease, a univariate analysis was performed using the chi‐square test and the Mann–Whitney test or the *t*‐test depending on the normal distribution of the continuous data. All significant variables from the univariate analysis were included in the logistic regression model. In the presence of complete separation (i.e., absence of outcome events in one subgroup), a Firth penalized logistic regression model was applied to reduce small‐sample bias and provide finite, more reliable estimates.

The results of the model were reported in full. In all analyses performed, a significance threshold for the *p* value of 0.05 was accepted. Statistical analysis was performed using StataMP v.18 statistical software.

## 3. Results

In the considered time period, 108 patients were found eligible from the patient registry of the Ce.Ma.R.S. outpatient clinic. In total, 12 patients were excluded from the study: six because they did not have a diagnosis of EDS or HSD and six because they were follow‐up exams rather than initial exams. Therefore, 96 patients were analyzed, of whom 51 underwent genetic testing as part of the routine diagnostic pathway. All patients were classified according to their clinical features based on the EDS criteria [6] with diagnoses of cEDS, vEDS, hEDS, and HSD established on the basis of clinical criteria and confirmed by genetic testing when available. These had a median age of 24 years (IQR = 28), 70.8% were female and median pain intensity was 3 (IQR = 4). The rate of patients that presented with moderate/severe pain symptoms was 49.0% (95% CI: 39.0–59.0), and in 83.1% of cases, the pain involved multiple body areas, with a higher frequency affecting the lower limbs, which was reported in 67.5% of cases. Pain in a joint with a single or recurrent dislocation episode was reported in 34.8% of patients. Regarding diagnosis, 31 patients (32.3%) had hEDS, 17 (17.7%) cEDS, 5 (5.2%) vEDS, and 43 (44.8%) HSD, and no other EDS subtypes were observed in the study population. Personal characteristics, diagnosis, specific clinical characteristics of the disease, and comorbidities are summarized in Table 1, where they are reported in relation to the overall sample, the group of patients with absent/mild pain symptoms, and the group with moderate/severe symptoms. By comparing these two patient groups, increasing age, female gender, the diagnosis of hEDS, the presence of multisite dislocations, and headache were factors associated with clinical presentations with moderate/severe pain. To address the issue of complete separation due to the absence of moderate/severe pain cases in the vEDS subgroup, a Firth penalized multivariable logistic regression model was used.

**TABLE 1:** Baseline characteristics of patients in relation to pain (no/mild pain vs moderate/severe pain).

Variables		Total *N* = 96	No/mild pain *N* = 49	Moderate/severe pain *N* = 47	*p* value
Characteristics of patients					
Age, 25°‐50°‐75° percentile (year)		13‐24‐41	8‐16‐27	21‐32‐46	0.001

Female, *n* (%)		68 (70.8)	30 (61.2)	38 (80.9)	0.034

Body mass index (BMI)^∗^, *n* (%)	Under	9 (9.7)	4 (8.5)	5 (10.9)	0.055
	Healthy	54 (58.1)	30 (63.8)	24 (52.2)	
	Over	23 (24.7)	11 (23.4)	12 (26.1)	
	Obesity	7 (7.5)	2 (4.3)	5 (10.9)	

Diagnosis, *n* (%)	EDS classical	17 (17.7)	8 (16.3)	9 (19.2)	0.001
	EDS vascular	5 (5.2)	5 (10.2)	0	
	EDS hypermobile	31 (32.3)	8 (16.3)	23 (48.9)	
	HSD	43 (44.8)	28 (57.1)	15 (31.9)	

Specific clinical features of disease					

Positive Beighton score^∗^, *n* (%)		52 (62.7)	28 (66.7)	24 (58.5)	0.444
Joint dislocation, *n* (%)	No	47 (49.0)	33 (66.7)	14 (29.8)	< 0.001
	Yes, one site	12 (12.5)	6 (12.5)	6 (12.8)	
	Yes, more sites	37 (38.5)	10 (20.4)	27 (57.5)	

Dislocation site, *n* (%)	Shoulder	29 (30.2)	8 (16.3)	21 (44.7)	0.002
	Jaw	9 (9.4)	5 (10.2)	4 (8.5)	0.776
	Knee/rotula	12 (12.5)	3 (6.12)	9 (19.2)	0.054
	Hip	12 (12.5)	1 (2.0)	11 (23.4)	0.002
	Finger/wrist	5 (5.2)	1 (2.0)	4 (8.5)	0.154
	Ankle	11 (11.5)	6 (12.2)	5 (10.6)	0.805

History of diagnosed hernias or pelvic organ prolapse, *n* (%)		34 (35.4)	14 (28.6)	20 (42.6)	0.152

History of surgery treatment, *n* (%)		17 (17.7)	10 (20.4)	7 (14.9)	0.479

Comorbidities					
Cardiovascular, *n* (%)		8 (8.3)	5 (10.2)	3 (6.4)	0.498
Headache, *n* (%)		24 (25.0)	6 (12.2)	18 (38.3)	0.003
Confirmed anxiety or depression disorders, *n* (%)		12 (12.5)	4 (8.2)	8 (17.0)	0.190
Diagnosis of fibromyalgia, *n* (%)		13 (13.5)	4 (8.2)	9 (19.2)	0.116
Neuropathy, *n* (%)		13 (13.5)	4 (8.2)	9 (19.2)	0.116

Abbreviations: EDS, Ehlers–Danlos Syndrome; HSD, hypermobility spectrum disorders.

^∗^Missing: 3 for BMI, 13 for Beighton score.

The penalized model (Table 2) confirmed and stabilized the findings of the standard logistic regression (Supporting Information S1), yielding consistent estimates. Firth penalized multivariable logistic regression (Table 2) confirmed that age, with each year increase corresponding to an OR of 1.06 (95% CI: 1.03–1.10), the diagnosis of hEDS with an OR of 6.25 (95% CI: 1.31–29.74), and headache with an OR of 3.97 (95% CI: 1.14–13.82) were statistically significant factors. The occurrence of dislocation affecting multiple sites had an OR of 3.06 (95% CI 0.94–10.01) with a *p* value of 0.064, whereas other factors such as gender, other diagnoses of EDS and HSD collected, the absence of dislocation, and dislocation in a single joint site did not show statistical significance.

**TABLE 2:** Multivariable logistic regression with Firth penalization for pain based on variables with statistical significance in the univariate analysis.

Variables		OR	95% CI		*p* value
			Lower limit	Upper limit	
Age		1.06	1.03	1.10	0.001

Female		1.13	0.34	3.75	0.846

Diagnosis	ESD classical	*Reference*			
	ESD vascular	0.05	0.00	2.04	0.115
	ESD hypermobile	6.25	1.31	29.74	0.021
	HSD	1.31	0.31	5.56	0.710

Headache		3.97	1.14	13.82	0.030

Dislocation	No	*Reference*			
	Yes, one site	0.62	0.13	2.93	0.543
	Yes, more sites	3.06	0.94	10.01	0.064

Concerning pain medication (Table 3), a statistically significant association (*p* < 0.001) was evident with pain, particularly highlighting how patients undergoing treatment with paracetamol and nonsteroidal anti‐inflammatory drugs were more likely to fall into the group of patients with moderate/severe pain. Targeted physiotherapy with strengthening exercises and pain treatment, as well as daily physical activity, did not show a significant association with pain.

**TABLE 3:** Pain treatment analysis.

Variables		Total *N* = 96	No/mild pain *N* = 49	Moderate/severe pain *N* = 47	*p* value
Pain relief medication, *n* (%)	No	46 (47.9)	39 (79.6)	7 (14.9)	< 0.001
	Yes	50 (52.1)	10 (20.4)	40 (85.1)	

Medication, *n* (%)	Paracetamol	21 (21.9)	3 (6.1)	18 (38.3)	< 0.001
	NSAIDs	22 (22.9)	4 (8.2)	18 (38.3)	< 0.001
	Opioids	6 (6.3)	1 (2.0)	5 (10.6)	0.082

General physical activity/other nonspecific exercise activities, *n* (%)		38 (39.6)	18 (36.7)	20 (42.6)	0.560
Specific strengthening exercises, *n* (%)		16 (16.7)	6 (12.2)	10 (21.3)	0.235

## 4. Discussion

Patients with a diagnosis of EDS or HSD exhibited particularly significant pain symptoms, with 49% of cases reporting moderate/severe pain. Considering a patient population affected by hEDS, Benistan et al. [13] reported a higher percentage of patients with moderate/severe pain, at 67% of cases. In the present study, examining only the population affected by hEDS, the percentage of patients with pain increased to 74%, a value that was in line with that reported by de Koning et al.[16], where this percentage was equal to 75% in a population of pediatric patients.

In the present study, the median pain intensity score was 3, which was lower than the pain score reported by Copetti et al. [15], where a sample of patients with EDS and HSD, classified with complex pathology, reported an average pain score of 6.5. This difference in pain intensity, in addition to the severity of the disease, may also be related to the demographic characteristics of the enrolled population: Copetti et al. [15] included patients with a mean age of over 35 years compared to the median age of 24 years in the present study, which included exclusively patients presenting for the first diagnostic exam.

The population analyzed in this study reported pain characterized by widespread involvement of multiple sites in the body, and only a smaller percentage of patients reported pain localized to a single specific joint. For this type of patients, previous studies have described a trend toward chronic pain progression over time with an increase in its intensity, potentially related to a condition of hyperalgesia and a central sensitization process of pain [22, 23]. Therefore, it becomes increasingly important to achieve an early diagnosis of EDS and HSD with a proper screening of patients in relation to their pain symptoms.

An increase in age, diagnosis of hEDS, and headaches were the three factors that identified patients most at risk for moderate/severe pain symptoms. The role of age is closely linked both to the course of the disease and its probable chronicity, as well as the diagnostic delay that often occurs, thus leading to inadequate patient management [11, 24]. Logistic regression data showed that each additional year of the patient’s age translated into a 6% increase in the risk of having a picture of moderate/severe pain. In a pediatric population of under 7 years old, De Koning et al. [16] showed that pain symptoms were a significant issue in 67% of children with a diagnosis of hEDS.

In the present study, a significant difference emerged between patients with hEDS compared to other forms of EDS and HSD.

Previous studies, including Aubry‐Rozier et al. [14], described a more severe clinical phenotype in hEDS compared with HSD, characterized by higher pain and disability levels. Nevertheless, these comparisons may be partially influenced by the diagnostic criteria themselves, since chronic musculoskeletal involvement represents one of the defining features of hEDS. Conversely, Copetti et al. [15] emphasized the relationship between pain severity and overall disease complexity, independently of the specific diagnostic classification. The intensity of pain was among the criteria used to make a differential diagnosis factors that differentiated between hEDS and HSD. In the present study, this diagnostic criterion might explain the difference in pain symptoms between patients diagnosed with hEDS and HSD as patients were enrolled at their first diagnostic exam. Headache was the third factor identified regarding pain with statistical significance.

Jari et al. [25] highlighted how for patients affected by hypermobility syndromes, the likelihood of suffering from headaches was 3.7 times higher compared to that of the healthy population among children and adolescents. Multiple regression of the present study showed that among patients affected by EDS and HSD who reported headaches, the risk of experiencing moderate/severe pain symptoms was four times higher than that of those who did not suffer from headaches. In general, headaches are one of the comorbidities described for patients affected by genetic disorders of connective tissue, along with depressive [26], neuropathic [27, 28], gastrointestinal [29], genitourinary [30], and cardiovascular conditions [31].

In the present study, patients suffering from psychiatric disorders, neuropathies, and a previous diagnosis of fibromyalgia were also identified, showing a higher frequency of cases in the group of patients with moderate/severe pain. This difference did not reach statistical significance, probably due to the low incidence of cases analyzed, but should still be taken into account when planning further in‐depth studies on larger samples.

Patients who had a history of dislocations involving multiple joint sites were found to have a 3.1 times higher likelihood of moderate/severe pain compared to patients who had not experienced dislocations, with a *p* value of 0.064. Although, from a statistical point of view, this variable does not reach significance, it is indeed consistent with the hypothesis that patients with episodes of dislocation affecting multiple joints may report more extensive tissue damage, particularly to proprioceptive joint receptors that are considered responsible for the worsening of pain symptoms [13]. Regarding the joints involved in dislocation, it was noted that dislocations affecting the shoulder and knee joints were associated with pain symptoms, while dislocations affecting the mandible did not have an association with moderate/severe pain.

The majority of patients presented with ongoing pain management therapy, and it is noteworthy that this group of patients reported more frequently moderate/severe pain symptoms. This finding likely reflects that patients with greater pain burden are more likely to require pharmacological treatment. However, the persistence of moderate/severe pain despite ongoing therapy may also suggest suboptimal pain control in a subset of patients. Of the 50 patients receiving pain management therapy, only six were taking opioid medications, whereas 21 were using only paracetamol. Previous studies have highlighted the potential need for opioids during acute pain and specific medication for neuropathic pain [32–35].

Several authors have highlighted the possibility of administering physiotherapy that would involve educational treatment and exercises to improve pain [14, 36, 37]. In the present study, only a few patients (16%) reported undergoing targeted physiotherapy treatment, thus showing the need to administer this treatment more widely and systematically in order to understand its true effectiveness [38].

### 4.1. Limitations

These results should be evaluated in light of the limitations that the study presented. First, the retrospective nature of the study resulted in some missing data that were not present in the patient’s medical record, such as the Beighton score, which had 13 missing entries. At the same time, the retrospective approach allowed for the examination of a larger sample of patients in their diagnostic phase. Second, it was not possible to follow up with the patients to assess the pain trajectory over time. Prospective and multicentric studies are necessary to evaluate the evolution of pain and to perform significant subgroup analyses. Finally, in the assessment of this group of diseases, a more detailed analysis of nonspecific symptoms is necessary, as they may play a significant role in relation to pain, as emerged regarding headaches.

A potential limitation of this study is the highly selected nature of the study population. As our institution is a referral center for rare skeletal diseases, most patients were referred with an already suspected diagnosis of hEDS/HSD. Therefore, the cohort may not fully reflect the broader population of patients evaluated in nonspecialized settings, potentially limiting the generalizability of the findings.

## 5. Conclusions

Patients with EDS or HSD present with significant pain symptoms from the early stages of their care. Increasing age, the diagnosis of hEDS, and a history of headaches are factors associated with moderate/severe pain symptoms that should be considered in the diagnosis of the disease. An early pain management strategy that includes a multidisciplinary treatment based on a pharmacological approach with opioid analgesics, targeted physiotherapy, and specific treatment for headaches should be promptly implemented, especially in patients with hEDS who are at a greater risk of developing moderate/severe pain.

## Author Contributions

This study was designed by Cristiana Forni, Morena Tremosini, and Luca Sangiorgi. The data were collected by Cristiana Forni, Mattia Morri, Alessia Di Cecco, and Maria Gnoli. The data were analyzed by Mattia Morri, Elena Pedrini, and Alice Moroni, and the results were critically examined by all authors. Mattia Morri had a primary role in preparing the manuscript, which was edited by Elena Pedrini, Alessia Di Cecco, and Luca Sangiorgi.

## Funding

This research was funded by the Ministry of Health, contribution 5xmille 2023 (incomes 2022) to Istituto Ortopedico Rizzoli, Grant No. 5M‐2023‐23687106.

## Disclosure

All authors have approved the final version of the manuscript and agree to be accountable for all aspects of the work.

## Conflicts of Interest

The authors declare no conflicts of interest.

## Supporting Information

Additional supporting information can be found online in the Supporting Information section.

## Supporting information


**Supporting Information** Supporting Information S1 presents the standard logistic regression model for pain (moderate/severe vs. absent/mild), including the same covariates considered in the main analysis.

## Data Availability

The data that support the findings of this study are available from the corresponding author upon reasonable request.
