# Supplementary material for: Characteristics of Pain in Patients Diagnosed With Ehlers–Danlos Syndrome and Hypermobility Spectrum Disorders. An Observational Study
Source: Pain Res Manag. 2026 Jun 27;2026:6664142. doi: 10.1155/prm/6664142 (PMC13309903; doi:10.1155/prm/6664142)
Supplement: Supplementary file 1 — Supporting Information Supporting Information S1 presents the standard logistic regression model for pain (moderate/severe vs. absent/mild), including the same covariates considered in the main analysis. [file PRM-2026-6664142-s001.docx]

**Supplementary material S1**. Multiple logistic regression of pain based on variables with statistical significance in the univariate analysis.

| **Variables** | | **OR** | **CI95%** | | **pvalue** |
| --- | --- | --- | --- | --- | --- |
|  |  |  | **Lower Limit** | **Upper Limit** |  |
| Age | | 1.07 | 1.03 | 1.11 | 0.000 |
| Female | | 1.20 | 0.33 | 4.39 | 0.785 |
| Diagnosis | ESD Classical |  |  |  |  |
|  | ESD Vascular | ------ |  |  |  |
|  | ESD Hypermobile | 7.98 | 1.49 | 42.77 | 0.015 |
|  | HSD | 1.39 | 0.30 | 6.44 | 0.678 |
| Headache | | 4.92 | 1.26 | 19.22 | 0.022 |
| Dislocation | No |  |  |  |  |
|  | Yes, one site | 0.56 | 0.10 | 3.02 | 0.499 |
|  | Yes, more site | 3.40 | 0.96 | 12.1 | 0.058 |
